# Evaluation of the risk factors for postoperative pectus excavatum and scoliosis in cystic lung disease

**DOI:** 10.1007/s00383-024-05925-4

**Published:** 2025-01-11

**Authors:** Tainaka Takahisa, Shirota Chiyoe, Sumida Wataru, Makita Satoshi, Amano Hizuru, Kano Yoko, Yasui Akihiro, Kato Daiki, Maeda Takuya, Gohda Yousuke, Ishii Hiroki, Ota Kazuki, Hinoki Akinari, Uchida Hiroo

**Affiliations:** Department of Pediatric Surgery, Nagoya University Graduate School of Medicine, 65 Tsurumai-Cho, Showa, Nagoya, Aichi 466-8550 Japan

**Keywords:** Cystic lung disease, Thoracotomy, Thoracoscopic surgery, Pectus excavatum, Scoliosis, Surgery

## Abstract

**Purpose:**

To analyze the frequency and predictive factors of the development of postoperative pectus excavatum and scoliosis in children who underwent surgery for cystic lung disease.

**Methods:**

This study examined patients who underwent surgery for cystic lung disease (open and thoracoscopic) between July 2000 and December 2018 with a > 3-year follow-up period. Lesion size, surgical outcomes, and subsequent musculoskeletal complications were compared between the open surgery and thoracoscopic surgery groups. Univariate and multivariate analyses were performed to identify predictive factors.

**Results:**

Overall, 90 patients (19 and 71 patients in the open and thoracoscopic groups, respectively) were included in this study. There was no significant difference in the incidence of pectus excavatum or scoliosis between open and thoracoscopic surgery; however, Haller’s index and Cobb angle were significantly higher in the open surgery group. In the univariate analysis, neonatal surgery and lesion size were substantial predictors of musculoskeletal malformations.

**Conclusion:**

Postoperative musculoskeletal deformities emerge after surgical treatment for cystic lung disease, with thoracoscopic surgery showing advantages in selected dimensions. Neonatal surgery and lesion size are pivotal prognostic factors for musculoskeletal complications. Further corroborative multicenter studies are imperative to substantiate these findings and foster enhanced patient outcomes.

## Introduction

Congenital cystic lung diseases include congenital pulmonary airway malformations, bronchopulmonary sequestration, bronchiectasis, bronchogenic cysts, congenital bronchial obstruction, and congenital lobar emphysema. Owing to the increased awareness of this medical condition and increased rate of prenatal diagnosis, the latest estimated incidence of congenital cystic lung disease is 1 in 2500 live births [1]. It is characterized by cystic and hamartomatous changes in the terminal airways, with a potential risk of recurrent chest infections and malignancy [1].

Due to advances in medical technology and equipment, thoracoscopic surgery has become an increasingly common surgical approach for cystic lung disease in recent years [2]. A systematic review of surgical procedures for cystic lung disease showed the advantages of thoracoscopic surgery over open thoracotomy; although the operative times are longer, it is associated with fewer complications and a shorter hospital stay [3]. However, there have been few reports on the long-term musculoskeletal outcomes [4]. The issue of musculoskeletal outcomes postoperatively for cystic lung disease may be the key to the advantages and disadvantages of this procedure.

Therefore, this study aimed to analyze the frequency and predictive factors of the development of postoperative pectus excavatum and scoliosis in children who underwent surgery for cystic lung disease in the long-term.

## Methods

### Study design and participants

Patients who underwent surgery (open or thoracoscopic) for cystic lung disease at our hospital between July 2000 and December 2018 and were followed-up at our hospital for > 3 years were included in the study. Open thoracotomy was performed as a conventional procedure, and the length of the chest wall incision was approximately the same as that of the skin incision. Thoracoscopic surgery was performed using four trocars (3 or 5 mm), and the lung was resected under completely thoracoscopic surgery. The resected lung specimen was collected in a bag and subsequently cut into small pieces/sections. Thoracic drains were placed in both procedures. Patients who underwent surgery in the neonatal period owing to poor respiratory status were also included. A retrospective review of the patient characteristics, lesion size, surgical outcomes (amount of blood loss and operative time), period to extubation postoperatively, postoperative complications, postoperative pectus excavatum, and postoperative scoliosis was performed.

The study protocol was approved by the hospital’s Medical Ethics Committee (approval number: 2023-0220).

### Assessment of surgical outcomes

**Fig. 1:**
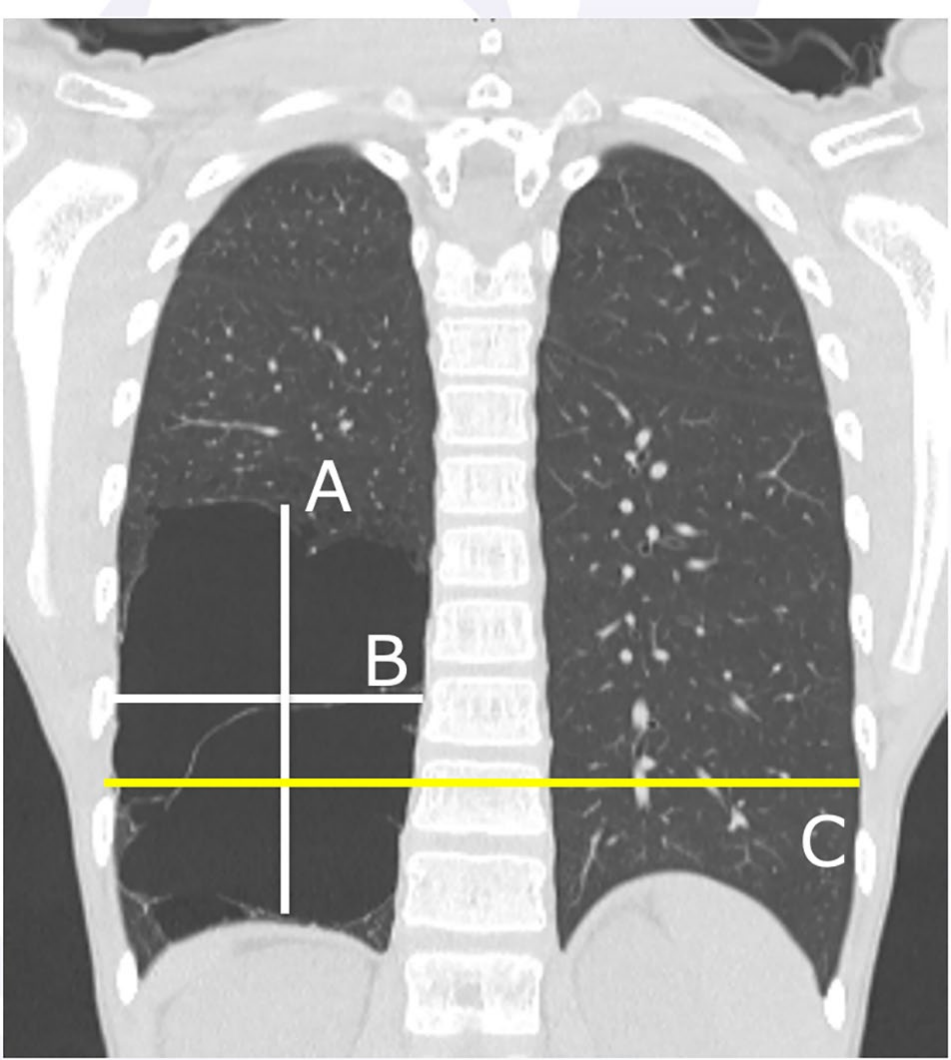
Lesion size: the longitudinal diameter (**A**, mm) × transverse diameter (**B**, mm) of the lesion to the unilateral thoracic diameter (1/2**C**, mm) on the coronal computed tomography (CT) section were measured

Lesion size was defined as the ratio of the longitudinal diameter × transverse diameter of the lesion to the unilateral thoracic diameter on the coronal computed tomography (CT) section (Fig. 1). Chest CT was performed when necessary, at the discretion of the outpatient physician. Haller’s index and the Cobb angle were measured by a blinded assessor using CT and plain radiographs, respectively. Pectus excavatum was defined as a Haller’s index > 3.0. Scoliosis was defined as a Cobb angle of ≥ 10° and was classified as light (10–20°), moderate (20–30°), or severe (≥ 30°) according to measurements using plain chest radiographs.

The patients were divided into the open (Op) and thoracoscopic (Th) surgery groups to compare surgical outcomes, postoperative complications, and the incidence of bronchial asthma, pectus excavatum, and scoliosis. Univariate analyses were performed to assess the influence of the following factors on the incidence of postoperative pectus excavatum and scoliosis: neonates, emergency surgery, lesion size (lesion area/thoracic diameter ratio), thoracoscopic surgery, and period to extubation. Multiple regression analysis was also performed to assess the influence of the following factors on the Haller index and Cobb angle: neonates, emergency surgery, lesion size (lesion area/thoracic diameter ratio), thoracoscopic surgery, and period to extubation.

### Statistical analysis

Birth weight and age were expressed as the median and range, and other quantitative values were expressed as the median and interquartile range. Qualitative variables were expressed as numbers and percentages (%). Statistical comparisons between both groups were performed using JMP Pro 16 software (SAS Institute Inc., Cary, NC, USA). Quantitative variables were analyzed using the Wilcoxon rank-sum test, and categorical variables were analyzed using the Chi-square test. A *p* value < 0.05 was considered as statistically significant and noted in bold in Table.

## Results

### Patient characteristics

Patient characteristics are summarized in Table 1. Overall, 90 patients (19 and 71 patients in the Op and Th groups) were included in this study. No significant differences were observed in sex, gestational age, or birth weight between both groups. Prenatal diagnosis was significantly more common in the Th group than in the Op group (50 [70%] vs. 6 [33%], respectively, *p* = 0.003). The mean weight at surgery was 7.1 kg and 6.5 kg in the Op and Th groups, respectively. There were no significant differences in preoperative history of pneumonia. Furthermore, the lesion size was significantly larger in the Op group than in the Th group (2.0 mm vs. 1.4 mm, *p* = 0.046). Emergency surgery was required in six (32%) and 10 (14%) patients in the Op and Th groups, respectively (*p* = 0.095). Lobectomy was the most common surgical procedure, with 18 (95%) and 65 (92%) patients in each group, respectively (*p* > 0.05). Patient age at the last outpatient visit was significantly higher in the Op group than in the Th group (12 vs. 6.9 years, respectively; *p* = 0.0001). The follow-up period was significantly longer in the Op group than in the Th group (7.8 vs. 5.9 years, respectively, *p* = 0.026).

**Table 1:** Characteristics of patients with congenital cystic lung disease

Characteristics	Op (*n* = 19)	Th (*n* = 71)	*p* value
Sex (male, %)	10 (53%)	33 (46%)	0.797
Gestational age (w, IQR)	38 (37–40)	39 (38–40)	0.498
Birth weight (g, range)	3.1 (0.97–3.86)	3.0 (2.7–3.2)	0.949
Cases of prenatal diagnosis (*n*, %)	6 (33%)	50 (70%)	**0.003**
Age at surgery (d, range)	322 (0–4422)	146 (0–5032)	0.839
Body weight at surgery (kg, IQR)	7.1 (3.1–19)	6.5 (4.0–8.1)	0.729
History of pneumonia (*n*, %)	6 (33%)	14 (20%)	0.351
Disease			0.523
CPAM	17 (89%)	56 (79%)	
Bronchial atresia	1 (5.2%)	6 (8.4%)	
Intralobular sequestration	1 (5.2%)	9 (13%)	
Lesion size			
Lesion/thorax ratio ([longitudinal diameter × transverse diameter] hemithorax diameter) (mm, IQR)	2.0 (1.7–2.9)	1.4 (1.0–2.2)	**0.046**
Emergency operation (*n*, %)	6 (32%)	10 (14%)	0.095
Operative procedure			0.754
Lobectomy	18 (95%)	65 (92%)	
Segmentectomy	1 (5.3%)	5 (7.0%)	
Partial resection	0 (0%)	1 (1.4%)	
Age at last outpatient visit (year, IQR)	12 (7.9–15)	6.9 (5.1–9.8)	**0.0001**
Follow-up period (years, IQR)	7.8 (5.7–12.0)	5.9 (4.0–8.3)	**0.026**

*Op*: Open group, *Th*: Thoracoscopic group, *IQR*, Interquartile range, *CPAM*, Congenital pulmonary airway malformation

### Surgical outcomes

Surgical outcomes are shown in Table 2. There was no significant difference in the operative time between the two groups; however, blood loss-to-weight ratio was significantly lower in the Th group than in the Op group. Early postoperative complications were not significantly different between both groups (2 [11%] vs. 10 [14%], *p* = 1.00), which included pneumothorax in five patients, pulmonary torsion in two, atelectasis in one, pyothorax in one case, and so on. Bronchial asthma was observed in three cases in the Th group, but there were no significant differences (*p* = 1.000). Musculoskeletal deformities included pectus excavatum in four (21%) and eight (11%) patients (*p* = 0.271), and scoliosis in two (11%) and two (2.8%) patients (*p* = 0.195), respectively. Operations (Nuss procedure) for pectus excavatum tended to be performed more frequently in the Op group than in the Th groups (3 [16%] vs. 2 [2.8%], respectively; *p* = 0.061). Haller’s index (2.55 vs. 2.41, respectively; *p* = 0.041) and Cobb angle (4.22º vs. 1.69º, respectively, *p* = 0.019) were significantly higher in the Op group than in the Th group.

**Table 2:** Surgical outcomes

	Op (*n* = 8)	Th (*n* = 14)	*p* value
Neonatal case (*n*, %)	8 (42%)	14 (20%)	0.069
Operative time (min, IQR)	200 (144–219)	211 (152–274)	0.452
Amount of blood loss (mL, IQR)	52 (15–114)	6.0 (2.0–40)	**0.0002**
Blood loss/body weight ratio (mL/kg, IQR)	6 (3.3–14)	1.3 (0.3–5.6)	**0.0003**
Period of intubation	0 (0–5)	0 (0–0)	0.243
Hospital days (days, IQR)	17 (11–30)	8 (6–12)	**0.0003**
Early postoperative complications (*n*, %)	2 (11%)	10 (14%)	1.000
Residual lesion (*n*, %)	1 (5.2%)	7 (9.9%)	1.000
Bronchial asthma (*n*, %)	0 (0%)	3 (4.2%)	1.000
Musculoskeletal deformities (*n*, %)	5 (26%)	11 (15%)	0.315
Pectus excavatum (*n*, %)	4 (21%)	8 (11%)	0.271
Operation	3 (16%)	2 (2.8%)	0.061
Haller index (ratio, IQR)	2.55 (2.44–3.38)	2.41 (2.25–2.63)	**0.041**
Scoliosis, total (*n*, %)	2 (11%)	2 (2.8%)	0.195
Light	1(5.3%)	1(1.4%)	
Moderate	1(5.3%)	0(0%)	
Severe	0(0%)	1(1.4%)	
Cobb angle (degree, IQR)	4.22 (1.21–6.25)	1.69 (0.49–2.98)	**0.019**

*Op* Open group, *Th* Thoracoscopic group, *IQR* Interquartile range

### Univariate and multivariate analyses

The univariate analysis results for the incidence of pectus excavatum and scoliosis are shown in Table 3. The unadjusted Odds ratio for factors affecting the incidence of pectus excavatum was 0.48 for thoracoscopic surgery, 0.73 for emergency surgery, 0.99 for lesion size, 1.00 for period to extubation, and 5.87 for neonates, which was significantly associated with neonatal surgery (*p* = 0.036). The unadjusted Odds ratio for the incidence of scoliosis was 1.02 for period to extubation, 2.63 for neonates, 3.65 for emergency surgery, 0.20 for thoracoscopic surgery, and 7.42 for lesion size, which was significantly associated with lesion size (*p* = 0.010).

**Table 3:** Univariable analysis for pectus excavatum and scoliosis

Univariable analysis for pectus excavatum		
	Unadjusted OR (95% Cl)	*p* value
Thoracoscopic surgery	0.48 (0.1–2.5)	0.376
Emergency operation	0.73 (0.1–5.8)	0.765
Lesion size (lesion area to thorax diameter ratio)	0.99 (0.4–2.1)	0.986
Period until extubation	1.00 (0.9–1.1)	0.957
Neonate	5.87 (1.1–31)	**0.036**

Univariable analysis for scoliosis		
	Unadjusted OR (95% Cl)	*p* value
Period to extubation	1.02 (0.9–1.2)	0.785
Neonate	2.63 (0.0–800)	0.737
Emergency operation	3.65 (0.1–165)	0.494
Thoracoscopic surgery	0.20 (0.0–4.6)	0.291
Lesion size (lesion area to thorax diameter ratio)	7.42 (0.79–70)	**0.010**

Multivariate analysis (multiple regression model) for the factors affecting the Haller index and Cobb angle is shown in Table 4. The only factor affecting the Haller index was non-thoracoscopic surgery (adjusted OR, 0.73 [95% CI 0.6–0.9]; *p* = 0.008). The only factor affecting the Cobb angle was lesion size (adjusted OR, 5.42 [95% CI 2.0–15]; *p* = 0.001).

**Table 4:** Multivariate analysis of the Haller index and the Cobb angle

Multivariate analysis of the Haller index (multiple regression analysis)		
	Adjusted odds ratio (95% Cl)	*p* value
Lesion size (lesion area to thoracic diameter ratio)	1.03 (0.8–1.4)	0.835
Period to extubation	1.24 (0.8–2.1)	0.394
Emergency	0.88 (0.7–1.1)	0.282
Neonate	1.17 (0.7–1.1)	0.097
Thoracoscopic surgery	0.73 (0.6–0.9)	**0.008**

Multivariate analysis of the Cobb angle (multiple regression analysis)		
	Adjusted odds ratio (95% Cl)	*p* value
Neonate	1.09 (0.3–4.4)	0.900
Period to extubation	1.01 (0.8–1.2)	0.890
Emergency	0.80 (0.1–5.4)	0.816
Thoracoscopic surgery	3.29 (0.9–13)	0.082
Lesion size (lesion area to thoracic diameter ratio)	5.42 (2.0–15)	**0.001**

*R*^*2*^ = 0.221, *R*^*2*^ = 0.204

## Discussion

There is ample evidence that thoracotomy in children is associated with an increased risk of developing musculoskeletal deformities, including chest wall deformities, such as cardiac, orbital, and scapular abnormalities, and scoliosis, with a prevalence rate of up to 50% [5–8].

The incidence of pectus excavatum was not related to the operative procedure; however, it was associated with neonatal surgery in the univariate analysis. Furthermore, the Haller index was associated with non-thoracoscopic surgery in the multivariate analyses. Non-thoracoscopic surgery was the most significant factor affecting the Haller index. As a prognostic factor, neonatal surgery was a result of the univariate analysis, which may be imprecise.

Scoliosis was associated with the lesion size but not with the operative technique. The Cobb angle was associated with thoracoscopic surgery, but thoracoscopy was not significant and was associated with lesion size in the multivariate analysis. The Cobb angle was associated with open thoracotomy based on a comparison between the two groups. However, multivariate analysis showed that non-thoracoscopic surgery was not significant and was associated with lesion size. The two factors may be related considering that larger lesions may have to be treated using open thoracotomy.

Previous reports have indicated that postoperative thoracic deformities occur in 14–30% of patients [4, 8–11]. Lawal et al. reported that postoperative thoracic asymmetry differed significantly between thoracoscopic and open surgery [9]. In contrast, Safa et al. found no significant difference in the incidence of musculoskeletal deformities between thoracoscopic and open thoracotomy [8]. Although there are few reports on postoperative thoracic deformities in cystic lung disease [4], a multicenter study in Japan found thoracic deformities in approximately 30% of patients [11]. In recent years, improvements in medical equipment and surgical techniques have enabled pediatric surgeons to perform minimally invasive surgeries on the thorax under various conditions; consequently, thoracoscopic surgery for cystic lung disease is now frequently performed [2]. A systematic review of short-term outcomes for cystic lung disease showed that thoracoscopic surgery is associated with fewer complications and shorter hospital stays than open thoracotomy [3].

Decreased intrathoracic pressure is another possible mechanism for deformities after pneumonectomy. This is observed in patients with congenital diaphragmatic defects, where lung hypoplasia on the affected side requires greater intrathoracic pressure due to reduced lung compliance [12], or even during upper airway obstruction in laryngomalacia [13]. Regarding the mechanism, the dead space in the thoracic cavity after the resection of a space-occupying lesion results in a negative intrathoracic pressure, which may deform the rib cage [10]. The most dramatic changes in the morphology of the sternum and thorax have been observed during childhood, when these structures ossify [14, 15]. Furthermore, the fragile thorax in neonates is more susceptible to deformation due to intrathoracic pressure than that in other age groups. The degree of ossification in neonates is the lowest among all developmental stages and may be responsible for the neonatal period being an independent risk factor for thoracic and spinal deformities after pneumonectomy [10]. Therefore, it was expected that patients with large cystic lesions and poor respiratory status requiring emergency surgery would be at a higher risk of developing pectus excavatum, especially during the neonatal period. In the present study, although emergency surgery was not listed as an incidence factor, neonates were listed as a risk factor for the development of pectus excavatum, which is consistent with previous reports.

Conventional thoracotomy involves dissection of the vastus lateralis and serratus anterior muscles, resulting in damage to the long thoracic nerve and atrophy of the serratus anterior muscle, which causes many sequelae such as scoliosis [5, 6]. A long-term evaluation after esophageal atresia surgery revealed different types of scoliosis [7]. Scoliosis has also been reported to occur equally in open vs. thoracoscopic lung resection [4], and occurs more frequently after thoracoscopic surgery (lung resection) than in the general population [16]. Therefore, it is possible that scoliosis after thoracotomy is a secondary result of rib fusion and pleural scarring [17, 18]. Further, different methods of chest closure, such as threading the ribs, may also have an impact. However, the Cobb angle of the thoracic spine was significantly greater in the Op group, indicating its influence. Multivariate analysis also revealed that lesion size was a factor influencing scoliosis and Cobb angle.

The limitations of this study include the following. First, this was a retrospective historical control study; therefore, postoperative management may have differed between both groups because the Op group was treated earlier. Moreover, the timing of surgery-included periods when different policies were in place. Second, there were differences in the follow-up periods, which may be due to differences in follow-up policies and prolonged follow-ups due to musculoskeletal deformities. Third, the number of included patients was small. This was due to the single-center nature of the study; however, a multivariate analysis was performed on several factors appropriate for the number of cases. A multicenter study with a large number of patients is desirable to assess the long-term musculoskeletal malformations after surgery for cystic lung disease.

In conclusion, the incidence of musculoskeletal deformities after cystic lung disease surgery was consistent with that reported in previous studies. The Haller index and Cobb angle were significantly higher in the Op group than in the Th group; however, the incidence of pectus excavatum and scoliosis was not significantly different. In the multivariate analysis, thoracoscopic surgery was a significant factor for a lower Haller index, and lesion size was a significant factor for a higher Cobb angle.

## Data Availability

No datasets were generated or analysed during the current study.
